# Outcomes of Conservative Management of Spontaneous Spinal Epidural Hematomas: A Retrospective Study

**DOI:** 10.7759/cureus.85430

**Published:** 2025-06-05

**Authors:** Kenichi Nitta, Hiroshi Imamura, Hiroshi Kamijo, Yasuaki Maeda, Momoko Uchida, Mayo Akita, Katsunori Mochizuki, Masao Hattori, Michitaro Ichikawa, Yuichiro Kashima

**Affiliations:** 1 Advanced Emergency and Critical Care Center, Shinshu University Hospital, Matsumoto, JPN

**Keywords:** clinical courses, conservative treatment, hospitalization, magnetic resonance imaging, spontaneous spinal epidural hematoma

## Abstract

Background: Spontaneous spinal epidural hematoma (SSEH) is a rare condition. The clinical characteristics and disease course of patients with SSEH treated conservatively for SSEH remain unclear. Therefore, this study aimed to clarify the clinical characteristics and courses of conservatively treated SSEH.

Methods: We retrospectively reviewed and analyzed patients with conservatively treated SSEH admitted to the Shinshu University Hospital between July 2009 and December 2023.

Results: We identified 22 patients with conservatively treated SSEH (9 men and 13 women) aged 40-85 years (median age, 69 years). A total of 13 patients had an American Spinal Injury Association (ASIA) score of E at admission. Seven patients were on antiplatelet therapy, and one was taking anticoagulants. The interval between the first and second magnetic resonance imaging (MRI) scans was five days (4-6.75 days). The size of hematomas decreased on the second MRI in all cases but did not regress. Patients were allowed to mobilize from bed after six days (5-7 days), with the length of hospital stay being 10.5 days (9-13 days), and no cases of recurrence were reported.

Conclusions: MRI re-evaluation approximately five days after the first assessment facilitated early rehabilitation in patients with conservatively treated SSEH. Consequently, conservative management should include neurological assessments and follow-up MRIs during hospitalization.

## Introduction

The occurrence of spontaneous spinal epidural hematoma (SSEH) is uncommon, with an estimated annual incidence of around 1 case per 1,000,000 people [1,2]. However, the advent of magnetic resonance imaging (MRI) has improved diagnosis and detection, leading to an apparent rise in the reported incidence of this condition [2,3]. While urgent surgical decompression and hematoma evacuation remain the mainstay of treatment for SSEH, an increasing number of patients managed conservatively for SSEH have been reported in the past decade [3-6]. Despite this, the standard conservative management of SSEH, including the optimal timing for re-evaluation and duration of bed rest, remains unclear. We hypothesized that in the absence of worsening neurological symptoms, repeating MRI on Day 5 and initiating rehabilitation if the spinal epidural hematoma decreased would be effective. Accordingly, this study aimed to test this hypothesis and clarify the clinical characteristics and progression of conservatively treated SSEH.

## Materials and methods

Study setting

This single-center retrospective observational study was conducted at a hospital equipped with an advanced emergency and critical care center in Nagano Prefecture, Japan. Patients with SSEH were transferred to this hospital from the scene or referred to other hospitals in Nagano Prefecture.

Study protocol

We retrospectively reviewed the medical records of patients with SSEH admitted to Shinshu University Hospital between July 2009 and December 2023. The diagnosis of SSEH was confirmed for all patients by both a radiologist and a neurosurgeon based on MRI findings. Spontaneous spinal epidural hematoma (SSEH) was defined as a spontaneously occurring hemorrhage located within the epidural space of the spinal column. Patients with SSEH due to postoperative or traumatic conditions, conditions associated with spinal punctures or catheters, or arteriovenous malformations were excluded [7,8]. Generally, patients with SSEH were evaluated using the American Spinal Injury Association (ASIA) Spinal Cord Injury Impairment Scale for neurological assessment [9]. In previous reports, conservative treatment was selected for cases showing neurological status improvement, minimal neurological deficits, or contraindications for surgery, including coagulopathy or the patient's general condition, and when patients declined surgery [3,4,10]. However, in this study, conservative treatment was only offered to patients with improving neurological status or minimal neurological deficits (ASIA grade D or E) [9]. Nonoperative cases were excluded because a delay in diagnosis meant that even if surgery had been performed, there would have been no improvement in the neurological symptoms. Moreover, patients with no or inadequate follow-up MRI scans during hospitalization were excluded from this study. This study was conducted with the approval of the Institutional Review Board at Shinshu University School of Medicine (approval number: 4355) and was conducted in accordance with the amended Declaration of Helsinki.

The data collected for this study included patient demographics (age and sex), comorbidities, an evaluation of the patients' general clinical status using the acute physiology and chronic health evaluation (APACHE) II score [11], and findings from MRI scans. The MRI data were used to identify the hematoma's central point and to calculate the total number of hematoma cases occurring at each spinal level. Furthermore, we recorded the clinical course and outcomes for each patient.

Patients conservatively treated for SSEH underwent urgent MRI evaluation if symptoms worsened after admission. If neurological symptoms improved or resolved, a follow-up MRI was performed on Day 5. Both the first and second MRI scans were further confirmed by at least one radiologist, with the attending physician confirming the findings for the follow-up scan. The second MRI evaluated the size and extent of the spinal epidural hematoma. Rehabilitation was initiated if the hematoma size had decreased after the second MRI scan. Bed rest continued until neurological symptoms improved, with a second MRI guiding further clinical decisions.

We used the modified Rankin Scale (mRS) as a functional assessment at both hospital admission and upon discharge or transfer to a rehabilitation institution. The mRS score ranges from 0 (no symptoms) to 6 (death) and measures levels of disability in conservatively treated patients with SSEH at discharge.

Statistical analysis

Continuous variables are summarized as medians with interquartile ranges (25th and 75th percentiles). All statistical analyses were performed using EZR software (developed by Saitama Medical Center, Jichi Medical University), which serves as a graphical user interface for R statistical software (version 2.13.0, published by the R Foundation for Statistical Computing, Vienna, Austria) [12]. EZR represents a customized version of R Commander (version 1.6-3), enhanced with statistical functions commonly employed in biostatistical research.

## Results

**Figure 1:**
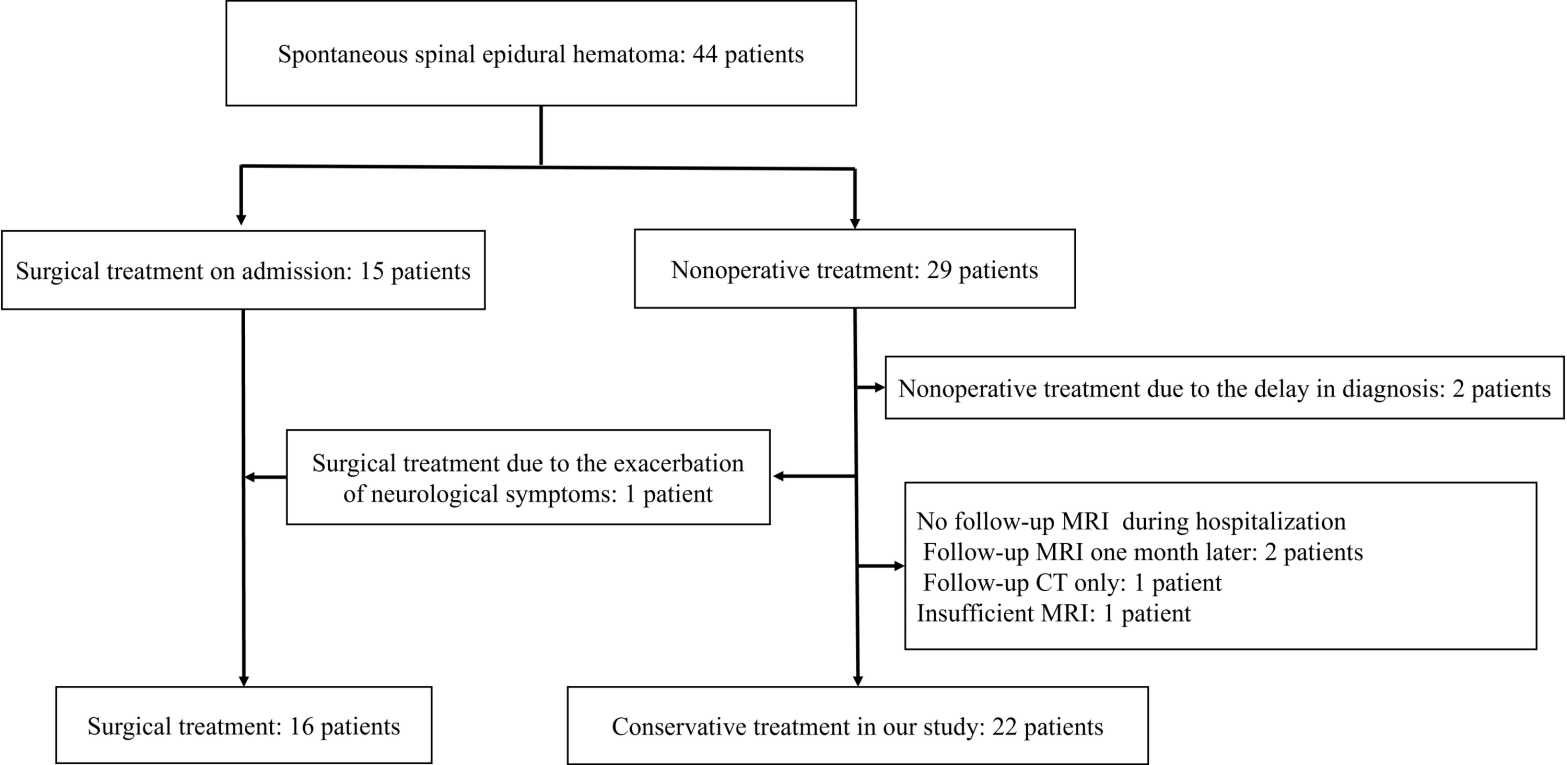
Flow chart of the study patients

A total of 44 patients with SSEH were admitted between July 2009 and December 2023. A total of 29 of these patients initially underwent nonoperative treatment. One patient underwent surgery 27 hours after being diagnosed with SSEH due to worsening neurological symptoms. Additionally, two patients were deemed unsuitable for surgery due to delayed diagnoses, which rendered symptom improvement post-surgery. After excluding four cases with insufficient MRI evaluations, 22 cases were included under conservative management (Figure 1).

As presented in Table 1, the median patient age was 69 years (interquartile range, 62-73 years); nine patients (41%) were male. A total of 11 of 22 patients (50%) were found to have hypertension. 

**Table 1:** Clinical and demographic characteristics of 22 patients conservatively treated for spontaneous spinal epidural hematoma Data are presented as median (interquartile range) or number (percentages). ASA PS, American Society of Anesthesiologists physical status; APACHE II, Acute Physiology and Chronic Health Evaluation II; ASIA, American Spinal Injury Association.

Characteristic	Value
Age (years)	69 (62–73)
Men	9 (41)
Comorbid diseases	
Hypertension	11 (50)
Diabetes mellitus	4 (18)
Hyperlipidemia	4 (18)
Coronary artery disease	1 (5)
General clinical status	
ASA-PS	2 (2‒2.75)
APACHE II score	8 (5‒9)
Prescription	
Antiplatelet drug	7 (32)
Anticoagulants	1 (5)
Pain at onset	
Neck	11 (50)
Back	9 (41)
Shoulder	7 (32)
Lower back	4 (18)
Initial ASIA grade	
C→D	2 (9)
D	7 (32)
E	13 (59)
Average length of hematoma	6 (5–7.75)
Number of involved segments	
3–4 segments	5 (23)
5–10 segments	15 (68)
>10 segments	2 (9)
Region of spinal hematoma	
Dorsal	
Right side	8 (36)
Central	5 (23)
Left side	8 (36)
Dorsal-ventral	1 (5)

Seven patients were on antiplatelet drugs, while one patient was taking anticoagulants. Among the 22 patients, 13 patients (59%) were classified as ASIA Grade E. 

MRI findings of SSEH

**Figure 2:**
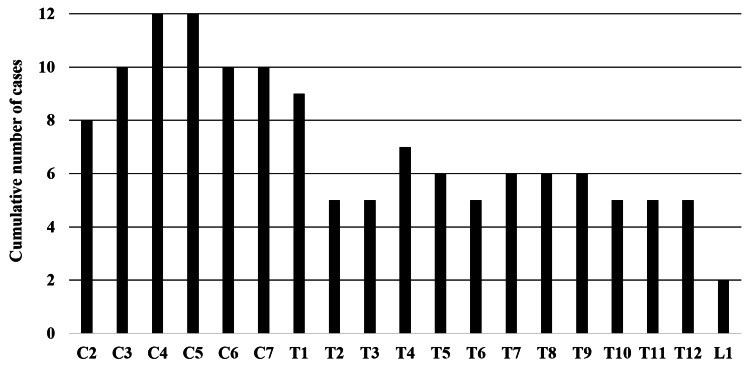
Numbers of hematoma cases. A bar graph illustrating the cumulative number of hematoma cases across different spinal levels.

Figure 2 illustrates the cumulative distribution of hematoma cases across different spinal levels.

**Figure 3:**
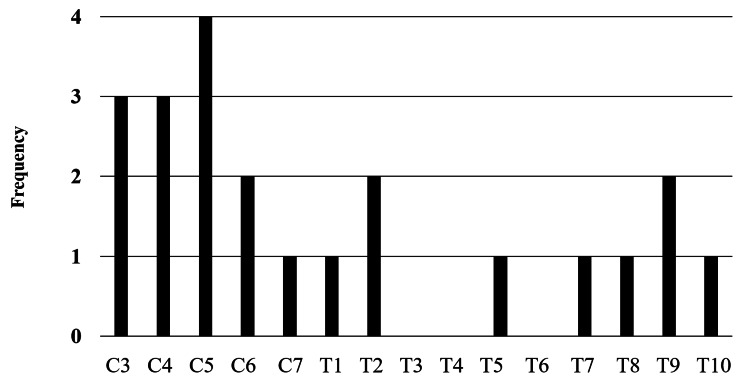
Central location of compression in the segmental distribution of all 22 hematomas.

The most frequent hematoma locations were observed at the fourth and fifth cervical vertebral levels, encompassing an average of six spinal segments. On MRI, the central point of the hematoma was located in the cervical region in 13 patients (59%) and the thoracic region in nine patients (41%) (Figure 3).

**Figure 4:**
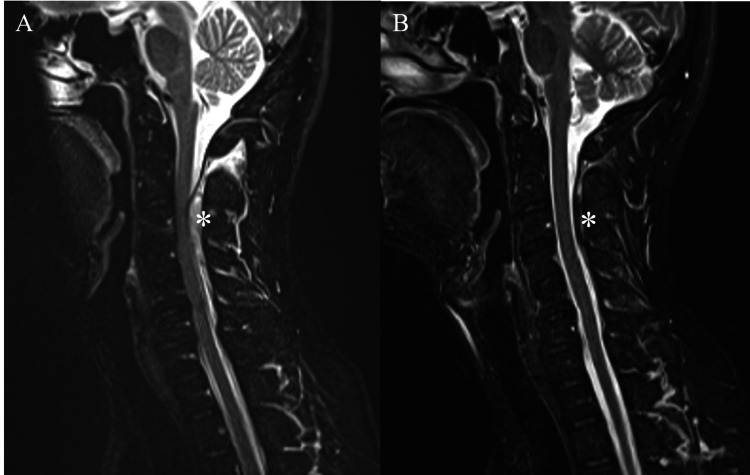
Hematoma in the case of conservative treatment (A) and (B) reveal the change of hematoma in a patient who has received conservative treatment. (A) A typical magnetic resonance imaging appearance of C2-C6 spontaneous spinal epidural hematoma (*) on sagittal T2-weighted imaging on admission. (B) The hematoma (*) decreased but did not resolve completely after five days of conservative treatment.

Spinal hematomas were mainly located in the dorsal region (Table 1). Further, in the second MRI scan, the radiologists evaluated that although the amount of spinal epidural hematoma decreased in all cases, it did not completely disappear. Additionally, Figure 4 shows typical MRI findings in the case of conservative treatment.

Clinical course, including treatments and rehabilitation

Tables 2-3 illustrate the management and outcomes of the 22 conservatively treated patients.

**Table 2:** Treatment and outcomes in 22 patients conservatively treated for spontaneous spinal epidural hematoma Data are presented as median (interquartile range) or number (percentage). ASIA, American Spinal Injury Association; MRI, magnetic resonance imaging.

Treatments and outcomes	Value
Treatments	
Antihypertensive drug	15 (68)
Nicardipine	14 (63)
Nifedipine	1 (4)
Analgesic	
Fentanyl	4 (18)
Morphine	2 (9)
Pentazocine	3 (14)
Acetaminophen	8 (36)
Loxoprofen	7 (32)
Tramadol	1 (4)
Steroid	3 (13)
Methylprednisolone	2 (9)
Dexamethasone	1 (4)
Outcomes	
Follow-up MRI period, day	5 (4–6.75)
Get out of bed, day	6 (5–7)
Hospital day	10.5 (9–13)
ASIA grade at discharge	
D	7 (32)
E	15 (68)

**Table 3:** Clinical presentation, management, and outcomes of 22 patients with conservative SSEH ASIA, American Spinal Injury Association; mRS, modified Rankin Scale; HT, hypertension; HL, hyperlipidemia; AP, angina pectoris; CI, cerebral infarction; DM, diabetes mellitus; CRF, chronic renal failure; AF, atrial fibrillation.

Patient No.	Age	Sex	Initial symptoms	Medical history	Antiplatelet agent or anticoagulant	Localization and extension	ASIA score (admission/discharge)	Second MRI day (days)	Release from bed rest (days)	Steroid	Anti-hypertensive drug	Analgesic	mRS (discharge)	Hospital stay (days)
1	40	F	Neck and back pain, paraparesis	None	None	Th2-9	D/E	4	5	None	None	Loxoprofen	0	8
2	45	M	Low back pain, paraparesis	None	None	Th7-12	D/E	5	6	None	None	None	0	10
3	67	F	Neck and back pain, tetraplegia	Colon polyp	None	C2-Th5	D/D	7	8	Methylprednisolone 1000 mg/day was administered on the first day, and the dose reduction was stopped for 4 days.	On the first day, the patient was given a nicardipine, which was changed to nifedipine the next day.	None	1	14
4	69	F	Orbital and back pain	Asthma, breast cancer (op.)	None	Th4-12	E/E	8	8	None	None	Pentazocine	0	13
5	80	F	Neck pain	None	None	C2-C5	E/E	5	6	None	None	None	0	9
6	85	F	Neck pain	CI	Clopidogrel	C2-C5	E/E	14	10	None	On the first day only, a nicardipine was administered.	Pentazocine	0	17
7	69	M	Neck and shoulder pain, hemiplegia	None	None	C3-Th1	C/D	7	7	None	On the first day only, nicardipine was administered	None	0	10
8	80	M	Chest and back pain	CI, gastric cancer (op.)	Clopidogrel	C7-Th4	E/E	2	5	None	None	None	0	8
9	74	M	Shoulder pain, paraplegia	CI	Clopidogrel	C3-Th7	C/D	5	8	None	The patient was administered nicardipine for 3 days.	Loxoprofen	0	16
10	70	M	Low back pain	CI, CRF	Clopidogrel	Th8-L1	E/E	2	6	None	None	Loxoprofen, acetaminophen	0	11
11	65	M	Shoulder pain	AP, duodenal ulcer	Aspirin	C2-C4	E/E	3	7	None	On the first day only, nicardipine was administered.	Fentanyl was administered for one day, then switched to loxoprofen.	0	9
12	58	F	Neck, shoulder and back pain	Cervical cancer (op.)	None	C2-C7	E/E	6	7	None	The patient was administered nicardipine for 2 days.	Morphine, loxoprofen	0	9
13	73	F	Neck and shoulder pain	Parkinson's disease	None	C2-Th1	D/D	7	8	None	The patient was administered nicardipine for 3 days.	Acetaminophen	4	20
14	43	F	Shoulder and back pain, hemiplegia	None	None	C2-Th4	D/D	2	3	None	None	None	1	6
15	55	M	Neck and back pain, numbness	Gout	None	C2-C6	E/E	5	3	None	On the first day only, nicardipine was administered	Loxoprofen	0	8
16	67	M	Neck pain, hemiplegia	None	None	C2-Th1	D/E	5	3	Dexamethasone 6.6 mg/day was administered for 3 days.	The patient was given a nicardipine for 4 days.	Pentazocine, acetaminophen	0	11
17	61	F	Neck pain, tetraplegia	HT, gout	None	C4-Th1	D/D	4	4	None	The patient was administered nicardipine for 4 days.	Fentanyl, morphine	1	9
18	81	F	Back pain	Gastric cancer (op.), Lumber spinal stenosis (op.)	None	Th4-11	E/E	7	7	None	The patient was administered nicardipine for 2 days.	Acetaminophen	0	13
19	69	F	Low back pain, paraparesis	HT	None	Th5-L1	D/D	6	6	None	On the first day only, nicardipine was administered	Acetaminophen, loxoprofen	0	13
20	73	M	Neck pain, hemiplegia	HT, DM, HL, internal artery stenosis (op.)	Clopidogrel	C4-C6	D/E	5	6	Methylprednisolone 250mg/day was administered for 1 day.	The patient was administered nicardipine for 2 days.	Fentanyl, acetaminophen	1	11
21	73	F	Neck and back pain, numbness	AF, internal carotid artery aneurysm (op.)	Aspirin, rivaroxaban	C5-Th1	E/E	6	6	None	On the first day only, nicardipine was administered	Fentanyl, acetaminophen	0	12
22	68	F	Neck and back pain, numbness	HT, sarcoidosis	None	C2-C6	E/E	4	4	None	Nifedipine	Acetaminophen, tramadol	0	5

Antihypertensive drugs, either intravenously or orally, were administered in 15 of 22 cases. Analgesics were required in 16 cases, and steroids were administered in three cases. In addition, hemostatic agents were administered in six cases. On the day of admission, one patient taking rivaroxaban had their medication reversed using freeze-dried human blood coagulation factor IX. Anticoagulants and antiplatelet agents were resumed after MRI confirmed a reduction in the hematoma size. 

The period between the first and second MRI scans was 5 (4-6.75) days. Patients resumed mobilization after 6 (5-7) days, and the median hospital stay was 10.5 (9-13) days. No patients experienced pain recurrence, neurological deficits, or rebleeding during hospitalization. Furthermore, during the follow-up assessment, no recurrence was observed in patients who underwent conservative treatment, both three months after hospital discharge and at the rehabilitation institution.

## Discussion

This study reviewed 22 cases of SSEH managed conservatively with MRI evaluations over 14 years. The interval between the first and second MRI scans was 5 (4-6.75) days. In all patients, hematomas on the second MRI decreased in size but did not completely resolve. Patients were mobilized after a median of 6 (5-7) days. The length of hospital stay was 10 (9-13) days. Our study provides valuable insights into the detailed conservative management of SSEH, a topic with limited detailed literature despite numerous case reports [3-9]. Previous reports indicate that 23-33% of SSEH cases are managed conservatively [5,13]. In this study, 59% of patients underwent conservative treatment for SSEH, and the reason for this high rate remains unclear. The widespread availability of MRI technology in Japan [14] has led to increased SSEH diagnoses, especially in minor cases, possibly explaining the higher rate of conservative management in this study than in previous reports.

Timings of the follow-up MRI evaluation and mobilization of patients treated conservatively for SSEH

Follow-up MRI is essential for monitoring hematoma resolution, with studies reporting varied imaging intervals [5,15-17]. Several reports have indicated that hematomas regress in the second MRI taken 25-31 days after onset [5,15,18]. However, Nagata et al. found that cervical SSEH required approximately 10 days for absorption, with a follow-up MRI performed at an average of 10.8 days post-admission [17]. Furthermore, they reported that neurological status improved before the hematoma resolved completely [17]. No study has explicitly delineated the optimal level and duration of rest during the acute phase of conservative management of SSEH. In this study, we re-evaluated the MRI after five days when no exacerbation of neurological symptoms or improvement was observed, and all patients had a reduction in spinal epidural hematoma size. Based on the results of the follow-up MRI, the patients were weaned and rehabilitated. However, it has been reported that patients should refrain from exercising within 10 days of onset because approximately 10 days are needed for SSEH to resolve on MRI [17]. Notably, this was the shortest time evaluated compared to any previous report, with no recurrence thereafter.

Conservative management in SSEH

Similar to intracranial hemorrhage, conservative treatment may include hemostatic agents, steroids, and antihypertensive therapy [18].

Administration of steroids may result in rapid improvement of the neurological deficit in SSEH [19,20], given that the pathophysiology of SSEH involves direct or vascular compression, causing progressive spinal cord edema and ischemia [17,19].

SSEH usually involves severe pain followed by spinal cord and nerve root compression symptoms [4]. Progressive motor and sensory deficits typically result in pain. Pain stimulates the sympathetic nervous system, resulting in increased blood pressure [21], and greater pain intensity correlates with an increased risk of developing delirium [22]. Pain management may be necessary in patients treated conservatively for SSEH. In our study, hyperacute pain was managed with continuous intravenous fentanyl as needed, followed by a transition to oral medications (acetaminophen, loxoprofen, and tramadol). However, if resistance to analgesia develops, there is a possibility of re-exacerbation of SSEH; therefore, care should be taken to re-evaluate patients with MRI, including neurological findings.

In our study, half of the patients treated conservatively received antihypertensive therapy. The association between hypertension and SSEH suggests that managing blood pressure may benefit from conservative treatment [23], though the direct necessity of antihypertensive therapy has not been explicitly detailed.

Antithrombotic agents in patients with SSEH on conservative therapy

Some authors have suggested that immediate replacement therapy in patients with coagulopathy prevents the progression of hematoma and improves neurological signs and symptoms without surgery [24,25]. In contrast, Connolly et al. [26] stated that coagulopathy-induced spinal bleeds are amenable to conservative treatment because the hematoma remains liquefied for a longer period than in cases with normal clotting time, thereby enabling the spread of the hematoma into the spinal epidural space. In our study, one patient receiving rivaroxaban underwent reversal therapy with prothrombin complex concentrate and resumed anticoagulant therapy a week later. Seven patients on antiplatelet agents did not require platelet transfusions during the perioperative period. Six patients resumed taking these medications during hospitalization, depending on their condition.

Recurrence of SSEH during hospitalization and after discharge

Yu et al. reported that conservative treatment could not prevent multiple episodes or rebleeding in patients [27], emphasizing the need for close monitoring to ensure that their neurological deficit does not worsen during the recovery period, which may necessitate prompt surgical intervention. In our study, only one patient underwent surgery 27 hours after being diagnosed with SSEH due to worsening neurological symptoms. Remarkably, conservative treatment requires repeated assessments of neurological findings and careful MRI observation.

Limitations

The present study has some limitations. First, its single-center retrospective design may introduce selection bias. Therefore, a multicenter prospective study is needed to confirm these findings. Second, the sample size (22 cases) reflects the rarity of the disease and may limit generalizability, especially given the homogenous patient population. Third, we hypothesized that if there were no worsening of neurological symptoms, the MRI would be repeated on Day 5 based on the experience of the spine surgeons. Fourth, excluding four cases with insufficient MRI evaluation may have influenced the results. Finally, detailed criteria for resuming anticoagulants and antiplatelet agents were not specified, raising concerns about the risk of rebleeding depending on the timing. Future research should focus on defining more specific optimal MRI re-evaluations and anticoagulation resumption protocols.

## Conclusions

This 14-year retrospective study of 22 cases provides valuable insights into the conservative management of SSEH, suggesting that a follow-up MRI on Day 5 and mobilization if the hematoma size decreases is an effective approach. Conservative treatment included pain control, antihypertensive therapy, and, in some cases, hemostatic agents and reversal of anticoagulation. Close monitoring of neurological status is crucial to promptly identify any worsening symptoms that may require surgical intervention.
